# Metagenomics and transcriptomics data from human colorectal cancer

**DOI:** 10.1038/s41597-019-0117-3

**Published:** 2019-07-05

**Authors:** Tina Visnovska, Patrick J. Biggs, Sebastian Schmeier, Frank A. Frizelle, Rachel V. Purcell

**Affiliations:** 1 School of Natural and Computational Sciences, Massey University, Auckland, New Zealand; 2 Present Address: Bioinformatics Core Facility, Oslo University Hospital Radium, Oslo, Norway; 3 School of Fundamental Sciences, Massey University, Palmerston North, New Zealand; 4 Department of Surgery, University of Otago, Christchurch, New Zealand

**Keywords:** Data publication and archiving, Colorectal cancer, Cancer genomics

## Abstract

Colorectal cancer is a heterogenous and mostly sporadic disease, the development of which is associated with microbial dysbiosis. Recent advances in subtype classification have successfully stratified the disease using molecular profiling. To understand potential relationships between molecular mechanisms differentiating the subtypes of colorectal cancer and composition of gut microbial community, we classified a set of 34 tumour samples into molecular subtypes using RNA-sequencing gene expression profiles and determined relative abundances of bacterial taxonomic groups. To identify bacterial community composition, 16S rRNA amplicon metabarcoding was used as well as whole genome metagenomics of the non-human part of RNA-sequencing data. The generated data expands the collection of the data sources related to the disease and connects molecular aspects of the cancer with environmental impact of microbial community.

## Abstract (summary)

**Table:** 

Design Type(s)	transcription profiling design • disease analysis objective
Measurement Type(s)	transcription profiling assay • rRNA 16S
Technology Type(s)	RNA sequencing • amplicon sequencing
Factor Type(s)	sex
Sample Characteristic(s)	human gut metagenome • Homo sapiens • colorectum

Machine-accessible metadata file describing the reported data (ISA-Tab format)

## Background & Summary

Colorectal cancer (CRC) is one of the most common types of cancer worldwide, in terms of both incidence and mortality^1^. Most cases of CRC are sporadic with no known genetic link. Environmental factors are therefore likely to play a critical role in the development of the disease, and a key characteristic of the colon is that it houses the largest proportion of the human microbiome, suggesting that this might play a role in causing CRC. Recent data points to the importance of the microbial communities in the gut, the microbiome, and possible links to the development of CRC^2–5^. If this is the case, understanding the role of the microbiome in CRC will have profound effects on cancer rates, since it is potentially relatively easily to manipulate, using diet, pre- and probiotics and faecal transplants^6–9^. However, despite the intense interest in the field and increasing evidence pointing to a role for the microbiome in CRC, convincing connections with clinical parameters and outcome are rarely seen.

CRC is a highly heterogeneous disease, with varying clinical outcomes, response to therapy, and morphological features, and molecular subtyping systems based on CpG-island methylation, microsatellite instability and gene mutations have shown strong associations with outcome and response to therapy in CRC^10–13^.

Contrary to other microbiome studies, where CRC is treated as a single disease entity, we focused on the association between Consensus Molecular Subtypes (CMS) of colorectal cancer and gut microbiome patterns in the accompanying primary publication^14^. We stratified a set of CRC tumour samples into CMS according to their gene expression profiles^15^ and assessed differences in bacterial communities among CMS. The gene expression profiles were generated using RNA sequencing, and 16S rRNA metabarcoding as well as metagenomic analysis of non-human portion of the RNA sequencing data were employed for bacterial taxa quantification. We analysed the enrichment/depletion of bacterial species in one subtype compared to the other subtypes and showed enrichment of certain oral bacteria associated with CMS, which was validated using targeted quantitative PCR.

The data generated in this study combine various views of each sample as multiple different methods were used to obtain information about the samples. This allows us to study associations between the results of the particular methods. Making the raw sequencing data available together with the scripts used for data processing and analysis, we enable reuse of the data and extend the collection of the data sources related to CRC, for which the aetiology is not yet well understood.

## Methods

**Fig. 1:**
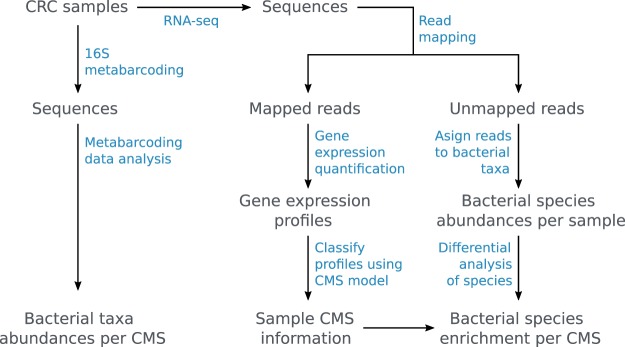
Workflow of sample and data processing. Samples and data are shown in grey and processes highlighted in blue.

Here, we present a more condensed version of the methods that led to data and analyses in the primary publication^14^. The workflow is shown in Fig. 1 and the names of the partial processes (depicted in blue in the figure) are used as titles in this section to structure the text. We make the raw sequencing data freely available in NCBI Sequence Read Archive^16^, and scripts together with more downstream analysis results are accessible as the Zenodo dataset^17^.

### Sample collection & handling

Tumour tissue was collected from 34 patients undergoing surgery for resection of colorectal tumours. None of the patients had received chemotherapy prior to surgery, and all patients provided written, informed consent. This study was carried out with approval from the University of Otago Human Ethics Committee (ethics approval number: H16/037). Table 1 shows patient metadata for the cohort. At the time of surgery, CRC tumour cores were taken and immediately frozen in liquid nitrogen and initially stored at −80 °C. They were subsequently transferred to RNAlater ICE^TM^ (Qiagen), and equilibrated for at least 48 hours at −20 °C, prior to nucleic acid extraction. RNA and DNA were extracted from 15–20 mg each of tissue using RNEasy Plus Mini Kit (Qiagen) and DNeasy Blood and Tissue Mini Kit (Qiagen), respectively. Tissue disruption was carried out using a Retsch Mixer Mill. RNA extraction included a DNAse treatment step, and DNA extraction included overnight incubation with proteinase K, and treatment with RNAse A. Purified nucleic acids were quantified using the NanoDrop 2000c spectrophotometer (Thermo Scientific, Asheville, NC, USA), and stored at −80 °C. Nucleic acids were extracted from all tumour samples in a single batch by one operator, to avoid inter-batch variation.

**Table 1:** Patient metadata for Predict colorectal cancer cohort. Gender categories M for male and F for female are used; column stage is post-operative Tumour-Node-Metastasis staging; U/C in CMS column stands for unclassified; and N/A in the side column stands for data not available.

SampleID	CMS	Age	Gender	Site	Side	Stage
CRC_01	CMS2	73	M	Colon	Left	1
CRC_02	U/C	62	F	Colon	Left	3
CRC_03	U/C	76	F	Colon	Right	2
CRC_04	CMS1	88	F	Colon	Right	2
CRC_05	U/C	68	F	Colon	Right	3
CRC_06	CMS3	63	M	Colon	Right	1
CRC_07	CMS2	81	F	Colon	Left	2
CRC_08	CMS2	74	M	Colon	Right	3
CRC_09	CMS1	83	F	Colon	Right	2
CRC_10	CMS2	81	M	Colon	Left	1
CRC_11	CMS3	79	F	Colon	Left	3
CRC_12	CMS3	79	F	Colon	Right	1
CRC_13	CMS2	74	F	Colon	Right	2
CRC_14	U/C	83	M	Colon	Left	2
CRC_15	CMS3	77	F	Colon	Right	3
CRC_16	CMS3	84	F	Colon	Right	3
CRC_17	U/C	77	M	Colon	Left	3
CRC_18	CMS2	58	M	Colon	Right	3
CRC_19	CMS2	77	M	Colon	Left	2
CRC_20	CMS3	74	M	Colon	Right	2
CRC_21	CMS1	75	F	Colon	Right	2
CRC_22	CMS3	78	F	Rectum	N/A	3
CRC_23	CMS2	78	F	Colon	Left	2
CRC_24	CMS3	45	F	Colon	Right	1
CRC_25	CMS1	78	F	Colon	Right	2
CRC_26	U/C	67	M	Colon	Right	3
CRC_27	CMS1	75	F	Colon	Right	3
CRC_28	CMS3	78	M	Colon	Left	1
CRC_29	CMS2	67	M	Colon	Right	2
CRC_30	CMS2	80	M	Colon	Left	2
CRC_31	CMS2	74	F	Colon	Right	2
CRC_32	CMS2	68	F	Colon	Left	4
CRC_33	CMS2	80	F	Colon	Right	3
CRC_34	CMS1	81	M	Colon	Right	3

### RNA-seq

Library preparation and ribosomal RNA depletion was carried out using Illumina TruSeq stranded total RNA library prep V1 and Ribo-Zero Gold. The ribosomal RNA depletion step has potentially removed a portion of bacterial ribosomal RNA alongside of the human one, hence losing some information on bacteria. However, the same method of depletion was used on all the samples thus the potential loss would effect all of them in a similar manner. RNA sequencing was carried out using the Illumina HiSeq. 2500 V4 platform, to produce 125 bp paired end reads. Each sample library was split equally to two HiSeq lanes and the sequences from the two lanes were merged for each sample during the data processing phase.

### Read mapping, Gene expression quantification, and Profile classification

Adapters and low quality segments were removed from the sequenced reads using fastq-mcf from EA Utils^18^ and SolexaQA++^19^. The cleaned reads were mapped to the GRCh38 reference human genome with STAR^20^ and the read count for each HAVANA annotated gene in every sample was calculated with htseq-count^21^. The read counts were transformed to gene expression profiles measured in transcripts-per-million (TPM) with DESeq2^22^. The published CMS classifier^15^ was used to assign a molecular subtype of the disease to each sample based on the gene expression profiles (for more details see^14^). We identified six samples as CMS1, 13 samples as CMS2 and nine samples as CMS3. No samples were classified as CMS4, and six samples were unclassified.

### Assignment of reads to bacterial taxa

A Kraken^23^ database was built containing all NCBI Refseq complete genomes or chromosome-level genomes (January 2017) and additional genomes of bacteria proposed to play a role in CRC, disregarding their genome status. The used bacterial genomes are listed in the files Supplementary_table_K1.xlsx (all complete and chromosome-level genomes) and Supplementary_table_K2.xlsx (of interest specifically for CRC) in the folder data/kraken of the accompanying repository. All RNA-seq reads that were not uniquely mapped to the human genome reference sequence were used as input to Kraken using this custom database for taxonomic classification per sample. Altogether, 2231 different bacterial species were detected in at least one sample and only 1.4% of the analysed reads were not assigned to any bacterial species. We visualised bacterial abundances per CRC subtype using Krona^24^ and the interactive plots are available at http://crc.sschmeier.com.

### Differential analysis of bacterial species in CMS

We analysed the enrichment/depletion of bacterial species in one subtype compared to the other subtypes employing a strategy similar to differential expression analysis. Using edgeR^25^, we identified bacterial taxa with considerable abundance differences among the subtypes. For each CRC sample we used the assigned CMS subtype, the list of identified bacterial species, and the read counts corresponding to the identified species as input data. We treated all samples of a certain CMS subtype as replicates belonging to the subtype and ran differential analysis of each CMS subtype against all the other classified samples. This analysis identified bacterial species that are enriched (or depleted) in a subtype as compared to all other subtypes. For further details regarding the analysis, please refer to the primary publication^14^.

### 16S rRNA metabarcoding

Libraries containing 16S rRNA were prepared with 20 ng of DNA for each sample using primer pairs flanking the V3 and V4 hypervariable regions of the 16S rRNA gene and Illumina sequencing adaptors and barcodes were added using limited cycle PCR. Amplicon sequencing was carried out using the Illumina MiSeq platform, and paired end reads of length 250 bp were generated.

### Metabarcoding data analysis

Short overlapping forward and reverse reads coming from the same fragment were joined together with FLASh^26^ to form sequences of the V3-V4 hypervariable 16S rRNA region. Afterwards, low quality regions were removed from the resulting fragments with SolexaQA++^19^. Microbiome analysis was carried out with the QIIME bioinformatics pipeline^27^ using the Greengenes database^28^ for taxonomy assignment. No further normalisation of the data was performed.

## Data Records

Sequenced genomic data from both RNA-seq and 16S rRNA metabarcoding experiments are stored in the Sequence Read Archive as the study SRP117763^16^. Data resulting from the analyses presented here are located in the folder data of the Zenodo repository^17^. The data are separated into several subfolders:

- The folder expr contains raw read counts in subfolder raw_counts, tpm-based expression profiles of all samples stored in file tpm.readyForClassifier.tsv and also file CMSclassifiedCRC.tpm.havana.tsv containing the CMS subtype classification. These files are the main outcomes of gene expression profile classifications.
- Results of the metagenomics analysis of the non-human genomic content of RNA-seq are located in folder kraken together with two tables (Supplementary_table_K*.xlsx) containing lists of bacterial species used in this metagenomics analysis.
- The folder 16S contains the biom file otu_table.biom resulting from the 16S rRNA metabarcoding analysis with QIIME and two partial abundance tables otu_table_sorted_*.txt.gz. The abundance tables are derived from the biom file and were used further for data visualisation in the primary publication as well as for the metagenomics method comparison.

## Technical Validation

### RNA-seq raw data quality

**Fig. 2:**
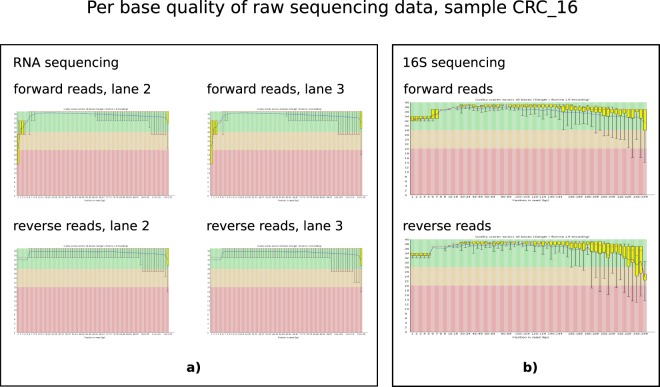
Per base quality of raw sequencing data, sample CRC_16. Output of FASTQC: (**a**) RNA sequencing, (**b**) 16S rRNA amplicon sequencing.

The quality of raw sequenced reads from RNA-seq experiments was assessed with FASTQC and was very good. A pair of representative per base quality plots of corresponding forward and reverse read pairs for one sample is shown in Fig. 2a). Regardless of the raw data quality, all the samples underwent routine data cleaning to ensure that no base was called with a Phred quality below 20. In Table 2, we show number of reads passing various data processing stages together with relative proportion of the reads passing two different stages.

**Table 2:** RNAseq, read counts and their ratios in various data processing stages for each sample. N/A in the CRC_14 sample stands for data not available.

sample ID	sequenced read pairs (count)	base quality ≥ 30 (in %)	cleaned read pairs (count)	cleaned in sequenced (in %)	uniquely mapped read pairs (count)	uniquely mapped in cleaned (in %)	fragments counted in expression profiles (count)	counted in mapped (in %)	read pairs for meta- genomics(count)	used for meta- genomics in cleaned (in %)
CRC_01	10210344	92.99	8196630	80.28	7150347	87.24	5301615	74.14	1046283	12.76
CRC_02	18195379	91.86	14099943	77.49	8953303	63.50	6339931	70.81	5146640	36.50
CRC_03	17060748	92.86	13695754	80.28	11763192	85.89	8737708	74.28	1932562	14.11
CRC_04	16113563	92.83	12984204	80.58	7771335	59.85	5515093	70.97	5212869	40.15
CRC_05	12283116	92.70	9787847	79.69	8177368	83.55	6141780	75.11	1610479	16.45
CRC_06	11889536	92.52	9444276	79.43	7689706	81.42	5485409	71.33	1754570	18.58
CRC_07	16767600	92.77	13384614	79.82	11174494	83.49	8282768	74.12	2210120	16.51
CRC_08	11692023	92.05	9148488	78.25	7211636	78.83	5370987	74.48	1936852	21.17
CRC_09	12414326	91.96	9744352	78.49	4853350	49.81	3473194	71.56	4891002	50.19
CRC_10	14196953	92.41	11216809	79.01	9659114	86.11	7307815	75.66	1557695	13.89
CRC_11	11891786	92.48	9384672	78.92	5842764	62.26	4172474	71.41	3541908	37.74
CRC_12	18376957	92.45	14448449	78.62	10438073	72.24	7535458	72.19	4010376	27.76
CRC_13	16869568	92.16	13310571	78.90	11664960	87.64	8747487	74.99	1645611	12.36
CRC_14	N/A	N/A	N/A	N/A	N/A	N/A	N/A	N/A	N/A	N/A
CRC_15	13680558	90.83	10481777	76.62	5713010	54.50	4035523	70.64	4768767	45.50
CRC_16	13982612	91.80	11035288	78.92	8867413	80.36	6509816	73.41	2167875	19.64
CRC_17	16873883	92.10	13306336	78.86	9959970	74.85	7181815	72.11	3346366	25.15
CRC_18	16663445	92.17	13179807	79.09	10641271	80.74	7400042	69.54	2538536	19.26
CRC_19	3518434	91.50	2721238	77.34	1258917	46.26	727030	57.75	1462321	53.74
CRC_20	13430061	91.98	10490785	78.11	1701669	16.22	1087471	63.91	8789116	83.78
CRC_21	9845344	90.87	7491472	76.09	5741211	76.64	4272125	74.41	1750261	23.36
CRC_22	15083803	91.89	11865373	78.66	10376744	87.45	7763574	74.82	1488629	12.55
CRC_23	9427192	90.64	7169010	76.05	5663964	79.01	4216223	74.44	1505046	20.99
CRC_24	11670754	90.49	8824150	75.61	6151634	69.71	4486074	72.92	2672516	30.29
CRC_25	15947939	92.42	12533528	78.59	8487630	67.72	6353442	74.86	4045898	32.28
CRC_26	14590462	91.97	11341012	77.73	9150589	80.69	6714043	73.37	2190423	19.31
CRC_27	14302258	92.33	11333614	79.24	10074723	88.89	7531937	74.76	1258891	11.11
CRC_28	11519270	91.74	9008972	78.21	7911036	87.81	5724147	72.36	1097936	12.19
CRC_29	10106322	93.12	8158472	80.73	7401313	90.72	5458587	73.75	757159	9.28
CRC_30	9323022	87.74	6502374	69.75	2697353	41.48	1445243	53.58	3805021	58.52
CRC_31	16617530	92.22	13095067	78.80	11255164	85.95	8326092	73.98	1839903	14.05
CRC_32	12418690	89.24	8994119	72.42	7557147	84.02	5644425	74.69	1436972	15.98
CRC_33	15556518	92.15	12165032	78.20	10928495	89.84	8206798	75.10	1236537	10.16
CRC_34	18455738	92.85	14793088	80.15	13219887	89.37	9995411	75.61	1573201	10.63

### 16S rRNA sequencing raw data quality

In Fig. 2b), we show quality of the 16S rRNA sequencing raw data for sample CRC_16. The other samples’ 16S rRNA quality plots looked similar. It can be seen that per base quality varied a little bit more along the 16S rRNA reads when compared to the RNA-seq reads, but overall the quality was very good for the 16S rRNA sequencing as well. Please note that the read length for the 16S rRNA sequencing was twice the read length of the RNA-seq, which together with differences between the used sequencing instruments explains differences in the quality plots. All the 16S rRNA samples underwent routine data cleaning to ensure that no base was called with a Phred quality below 20. In Table 3, we show number of reads passing various data processing stages together with relative proportion of the reads passing two different stages.

**Table 3:** 16S rRNA metabarcoding, read counts and their ratios in various data processing stages for each sample.

sampleID	sequenced read pairs (count)	base quality ≥ 30 (in %)	cleaned fragments (count)	cleaned in sequenced (in %)
CRC_01	333335	89.75	176823	53.05
CRC_02	238221	92.15	126462	53.09
CRC_03	356650	91.98	187474	52.57
CRC_04	307676	92.35	165991	53.95
CRC_05	261798	93.33	148547	56.74
CRC_06	122630	92.36	67416	54.98
CRC_07	175589	94.39	104310	59.41
CRC_08	210849	93.22	119255	56.56
CRC_09	238258	94.06	133700	56.12
CRC_10	233536	92.30	129813	55.59
CRC_11	291890	87.52	148406	50.84
CRC_12	173621	93.14	96744	55.72
CRC_13	204471	92.43	113588	55.55
CRC_14	255851	92.52	141822	55.43
CRC_15	254700	93.37	145899	57.28
CRC_16	210014	94.06	126141	60.06
CRC_17	197765	92.96	110784	56.02
CRC_18	161324	92.88	90441	56.06
CRC_19	147498	93.66	82425	55.88
CRC_20	235318	92.33	127779	54.30
CRC_21	169421	93.64	96627	57.03
CRC_22	249364	93.55	144146	57.81
CRC_23	171152	91.54	91281	53.33
CRC_24	102066	91.90	54880	53.77
CRC_25	334496	93.67	195656	58.49
CRC_26	265504	93.22	150713	56.76
CRC_27	69391	93.02	40037	57.70
CRC_28	137873	91.43	74333	53.91
CRC_29	176936	94.06	107348	60.67
CRC_30	202971	94.46	118078	58.17
CRC_31	220216	93.44	126807	57.58
CRC_32	108880	93.34	61688	56.66
CRC_33	219198	94.42	128793	58.76
CRC_34	305438	93.88	178759	58.53

## ISA-Tab metadata file

Download metadata file

## Data Availability

All the code used to process the genomic data is freely available as a part of the provided Zenodo repository^17^ and the code is located in the folder named scripts. The scripts folder also contains dependencies listed in the file used_packages_and_their_versions.tsv and the used parameter values listed in used_parameters.tsv. Depending on the scripts’ functionality, they are separated into various folders: The folder rnaseq-subtype-classification contains scripts used for read mapping, gene expression quantification, and profile classification. The folder kraken/human-unmapped contains scripts to assign reads to bacterial taxa. The folder kraken/diff-expr-taxa contains scripts for differential analysis of bacterial species in CMS. The folder 16S-metabarcoding contains scripts for metabarcoding data analysis.
